# Prostacyclin reverses platelet stress fibre formation causing platelet aggregate instability

**DOI:** 10.1038/s41598-017-05817-9

**Published:** 2017-07-17

**Authors:** M. Z. Yusuf, Z. Raslan, L. Atkinson, A. Aburima, S. G. Thomas, K. M. Naseem, S. D. J. Calaminus

**Affiliations:** 1 Centre for Atherothrombosis and Metabolic Disease, Hull York Medical School, University of Hull, Hull, HU6 7RX UK; 2 Institute of Cardiovascular Sciences, College of Medical and Dental Sciences, University of Birmingham, Birmingham, B15 2TT UK; 3 Centre of Membrane Proteins and Receptors (COMPARE), Universities of Birmingham and Nottingham, Midlands, UK

## Abstract

Prostacyclin (PGI_2_) modulates platelet activation to regulate haemostasis. Evidence has emerged to suggest that thrombi are dynamic structures with distinct areas of differing platelet activation. It was hypothesised that PGI_2_ could reverse platelet spreading by actin cytoskeletal modulation, leading to reduced capability of platelet aggregates to withstand a high shear environment. Our data demonstrates that post-flow of PGI_2_ over activated and spread platelets on fibrinogen, identified a significant reduction in platelet surface area under high shear. Exploration of the molecular mechanisms underpinning this effect revealed that PGI_2_ reversed stress fibre formation in adherent platelets, reduced platelet spreading, whilst simultaneously promoting actin nodule formation. The effects of PGI_2_ on stress fibres were mimicked by the adenylyl cyclase activator forskolin and prevented by inhibitors of protein kinase A (PKA). Stress fibre formation is a RhoA dependent process and we found that treatment of adherent platelets with PGI_2_ caused inhibitory phosphorylation of RhoA, reduced RhoA GTP-loading and reversal of myosin light chain phosphorylation. Phospho-RhoA was localised in actin nodules with PKA type II and a number of other phosphorylated PKA substrates. This study demonstrates that PGI_2_ can reverse key platelet functions after their initial activation and identifies a novel mechanism for controlling thrombosis.

## Introduction

In healthy blood vessels platelets are exposed to endothelial derived nitric oxide (NO) and prostacyclin (PGI_2_) which act to inhibit platelet activation^1^. However, upon vascular damage platelets overcome this inhibition allowing the formation of a thrombus. Within the thrombus, the activation status of the platelet is determined by the relative activatory and inhibitory signalling in the microenvironment of the vascular lesion and its location within the thrombus. Here, fully activated platelets are found within a core region, while those at the periphery are only weakly activated^2^. However, the diffusion of platelet agonists within the thrombus is not uniform^3^, and furthermore there is little known on the diffusion of the endogenous platelets inhibitors, NO and PGI_2_, within the thrombus. Furthermore as endothelial release of PGI_2_ is induced by the activity of thrombin^4^, this brings forward the idea that this induction of PGI_2_ by thrombin is not only limited to the inhibition of non-stimulated platelets, but also to reverse the activation of activated platelets within the thrombus in order to effectively control the extent of the thrombus formation induced by the injury.

In order for platelets to be able to form thrombi effectively within the high shear environment of the vasculature, platelet adhesion at sites of vascular damage leads to significant remodeling of the actin cytoskeleton. The reorganization of the actin cytoskeleton is a complex process requiring co-ordinated modulation of actin polymerization, in order to drive the sequential formation of filopodia, actin nodules, lamellipodia and stress fibres (reviewed in ref. 5). Individual members of the RhoGTPase family have been implicated in this progessive remodeling of the cell architechture. Cdc42 drives filopodia formation^6^, Rac activation of WASP and the Arp2/3 complex generates lamellipodia^7^, and RhoA activity drives stress fibre formation^8^. At present it is unclear which RhoGTPase is responsible for actin nodule formation, although Rac has been identified to be present within the nodule^9^. This reorganization is dynamic, requiring constant signaling in order to prevent lamellipodial collapse^10^. Defective cytoskeleton remodelling leads to platelet spreading defects, reduction in thrombus size and an increase in thrombus instability^8, 11–13^.

Understanding the role of PGI_2_ is therefore critical to understand platelet function. PGI_2_ causes platelet inhibition via binding of PGI_2_ to the IP receptor, leading to the activation of adenylyl cyclase, and the synthesis of cyclic adenosine 3′, 5′ monophosphate (cAMP). Elevated cAMP activates PKA, the primary effector of cAMP signalling in platelets, leading to the inhibition of platelet functions *in vitro* and diminished platelet accrual at sites of vascular injury *in vivo* ^1^. Once activated PKA isoforms blunt multiple aspects of platelet function including Ca^2+^ mobilisation, integrin activation, secretion, although knowledge of how these specific platelet functions are targeted by cAMP signalling remains unclear^14–16^. Proteomic studies have demonstrated that cAMP/PKA signalling targets a cluster of potential proteins associated with cytoskeletal reorganisation suggesting that the cytoskeleton is a major target in order to effectively mediate platelet regulation^17^. In this context, we have recently described a mechanism by which cAMP signalling prevented platelet shape change through inhibition of RhoA signalling and phosphorylation of myosin light chains (MLC)^18^. However, the mechanisms by which cyclic nucleotide signaling can return platelets to the resting state after activation, thereby preventing permanent attachment to the thrombus or at points distant to the injury, are unknown.

In the present study we examined the role of cAMP in modulating the function of activated platelets. We found that cAMP signaling can reverse stress fibre formation, significantly reduce the surface area coverage of the platelet, and induce actin nodule formation in a cAMP and PKA dependent manner. This reversal of stress fibre formation, and surface area coverage was associated with inhibitory phosphorylation and inactivation of both RhoA and the localisation of pRhoA to actin nodules. Furthermore these changes in the actin cytoskeleton were associated with a reduction in surface area coverage in platelets flowed over fibrinogen in high shear environment. This data identified that PGI_2_ can reverse platelet activation leading to alteration of the platelets function in a high shear environment.

## Results

### Effect of PGI_2_ on Platelet aggregate formation on fibrinogen

**Figure 1:**
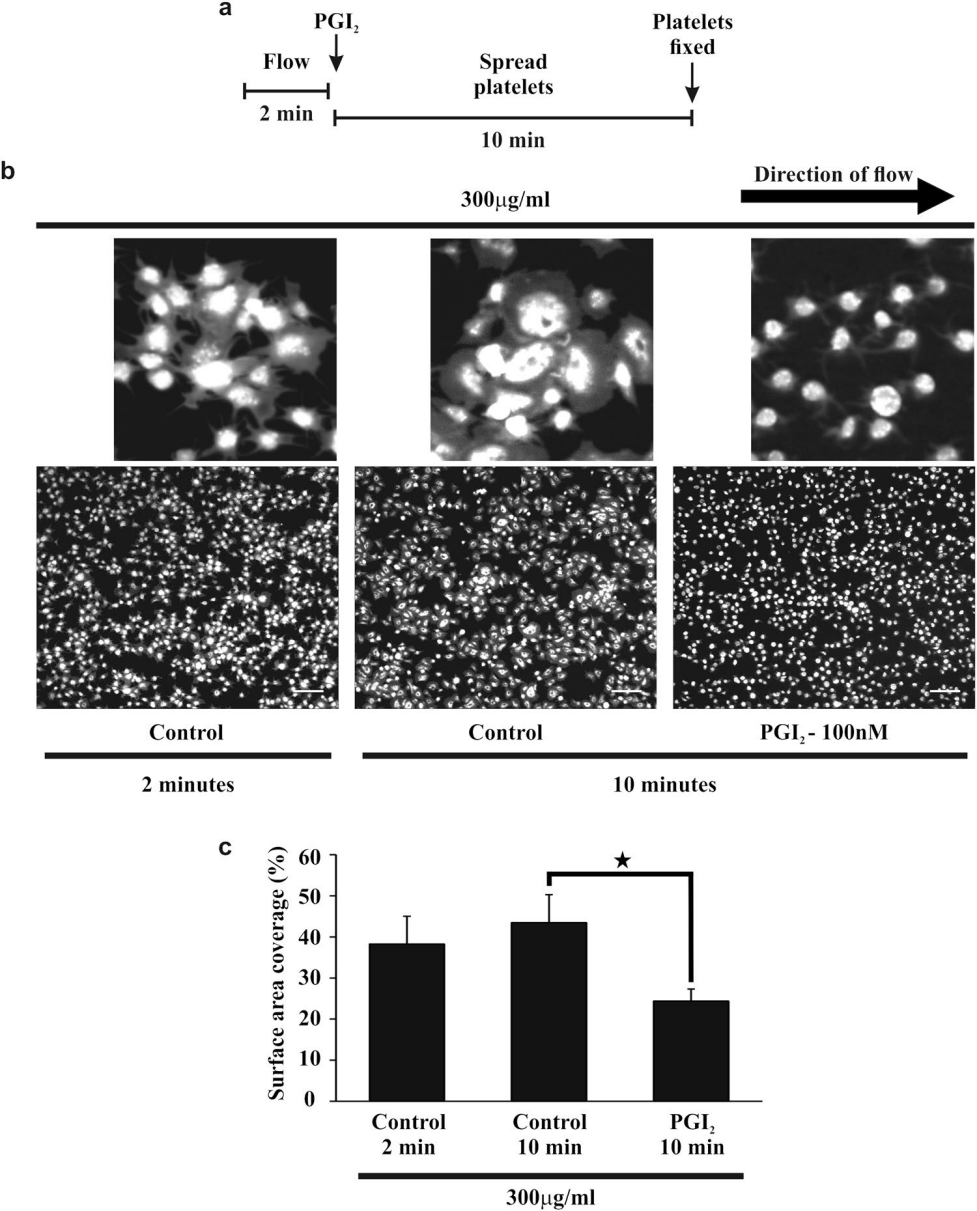
Post perfusion of 100 nM PGI_2_ induces embolisation of performed thrombi on fibrinogen. Whole blood, stained with 10 μM DiOC_6_ was flowed over fibrinogen (300 μg/ml) coated slides for 2 minutes at a shear rate of 1000 s^−1^, to enable the formation of thrombi. After 2 minutes either tyrodes alone or tyrodes containing 100 nM PGI_2_ were perfused over the preformed thrombi for 10 minutes at 1000 s^−1^. (**a**) Schematic of the experimental condition of the flow. (**b**) Representative images of the thrombi observed under these different experimental conditions. Scale bar is 20 μm. The insert shows an enlarged image of their respective images with a scale bar of 5 μm. (**c**) The surface area of the thrombi at 2 minutes of flow and after 10 minutes of perfusion with 100 nM PGI_2_. Thrombi were fixed with 4% paraformaldehyde, before restaining with 10 μM DiOC_6_ overnight. Thrombi were then imaged using flourescent microscopy, and analysed using ImageJ to obtain the surface area. Analysis completed from n = 3 experiments. p < 0.05.

Previously we have shown that blood preincubated with PGI_2_ (100 nM) can inhibit thrombus formation on fibrinogen^19^. However the effects of PGI_2_ on activated platelets has not been shown. Therefore, whole blood was flowed over fibrinogen (300 ug/ml) at arterial rates of shear (1000 s^−1^) resulting in the formation of small thrombi and a surface area coverage of 38.2 ± 6.8% (Fig. 1 and Supplementary Video. 1). Perfusion of these thrombi with PGI_2_ (100 nM) led to a significant reduction in surface area coverage to 24.4 ± 2.9% (p < 0.05). In contrast, perfusion of the thrombi with buffer alone had no significant effect on surface area coverage (Fig. 1a,b and Supplementary Videos 2 and 3). Further analysis of the platelets identified that there had been a noticable reduction in surface area in the spread platelets, indicating that PGI_2_ may reverse platelet spreading on fibrinogen.

### PGI_2_ reverses stress fibre formation in spread platelets on fibrinogen

**Figure 2:**
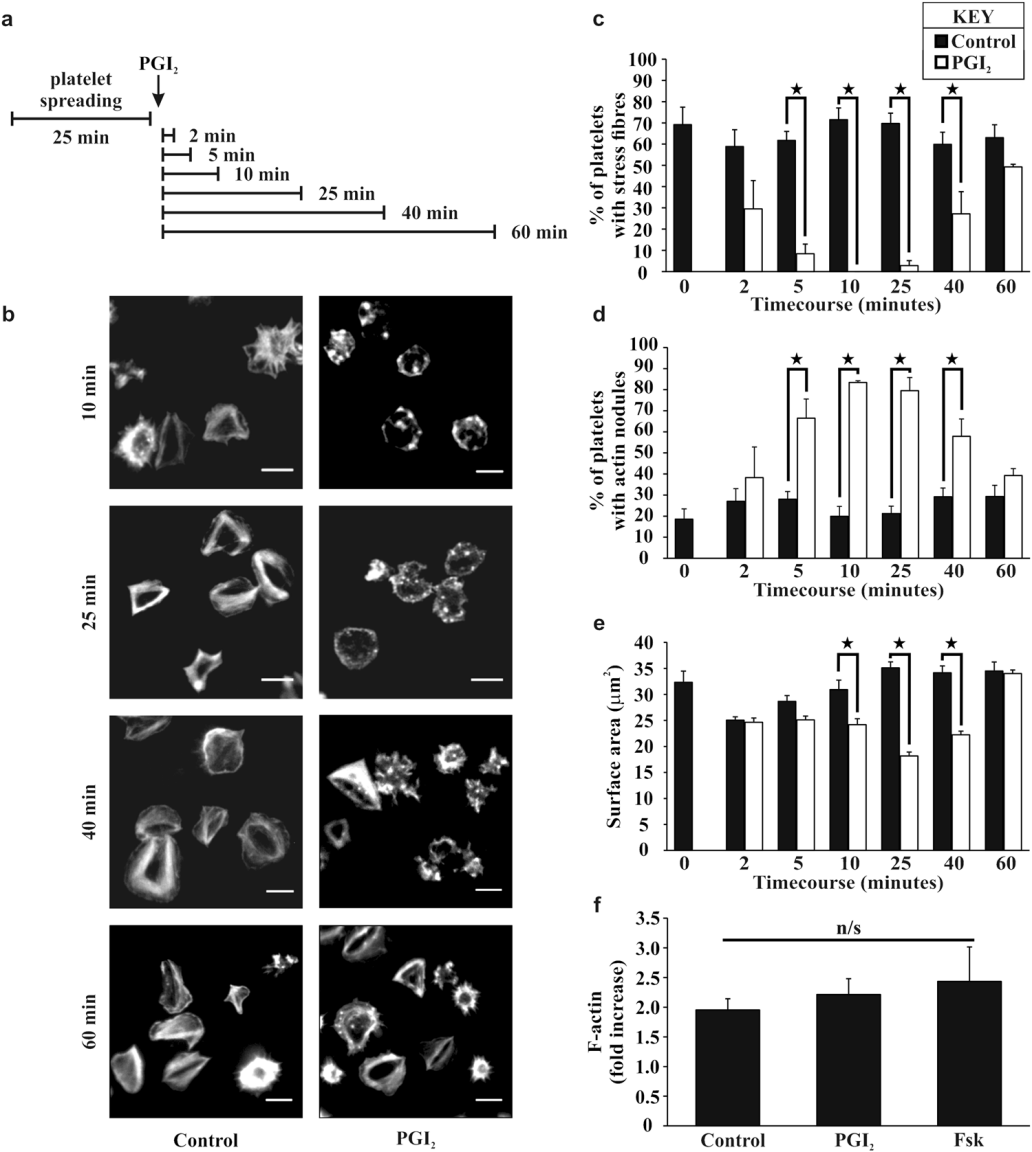
Post treatment of PGI_2_ induces stress fibre reversal and actin nodule formation in platelets spread on fibrinogen in a time dependent manner. Platelets (2 × 10^7^/ml) were spread on 100 μg/ml fibrinogen for 25 minutes, washed with PBS, and then 10 nM of PGI_2_ was added, for a further 2–60 minutes as per the representative experimental design in part (**a**) The platelets were then fixed and stained with FITC-phalloidin before being imaged via fluorescent microscopy. (**b**) Representative images of each condition of the experiment. (**c**) The number of spread platelets containing stress fibres was identified in control and PGI_2_ treated samples. (**d**) The number of spread platelets containing actin nodules was identified in control and PGI_2_ treated samples. (**e**) The average surface area of the spread platelets was analysed for each timepoint in control and PGI_2_ treated samples. Analysis was performed using Image J. The experiments are an average of n = 5. p < 0.05. Scale-bar 5 μm. (**f**) Platelets were spread as above for 25 minutes and then treated for 10 minutes with either 10 nM PGI_2_ or 1 μM forskolin. Assessment of the filamentous actin was performed using the F-actin assay. The experiments are an average of n = 3.

Analysis of platelet spreading in the absence of PGI_2_ demonstrated within 5 minutes of adhesion, the majority of platelets had formed actin nodules, but by 25 minutes that these actin nodules had been replaced by platelets containing stress fibres (Supplementary Figure S1). These stress fibres were maintained for up to 45 minutes post adhesion (longest time tested) which is consistent with previously published data^9^. The actin cytoskeleton has been implicated in thrombus stability and is known to be modulated by PKA signaling in multiple cell types^20–22^. Therefore, in the next series of experiments PGI_2_ (10 nM) was added to the spread platelets after 25 minutes, and spreading evaluated for a further 60 minutes (Fig. 2a). In the absence of PGI_2_ the number of platelets with stress fibres did not alter appreciably over this timecourse. In contrast the presence of PGI_2_ caused a rapid loss of actin stress fibres in spread platelets. This effect was maximal at 10 minutes post PGI_2_ treatment before returning to control levels after 60 minutes (Fig. 2b,c). Interestingly the loss of stress fibres was associated with the reciprocal appearance of circular dot like F-actin rich structures, which was again maximal at 10 minutes before returning to control values by 60 minutes (Fig. 2b,d). The F-actin circular structures induced by PGI_2_ were confirmed to be actin nodules, via identification with immunochemistry of the presence of actin nodule markers; the Arp2/3 complex, pan-pTyrosine substrate and WASP (Supplementary Figure S2a,b and data not shown)^9^. The alterations in actin structure was associated with a dynamic change in platelet surface area. It was observed that as the actin structures rearranged the platelet surface area significantly contracted, but expanded again as the actin stress fibres reformed (Fig. 2b,e). Interestingly, the change in actin structures had no effect upon the number of adherent platelets (Supplementary Figure S3). In order to identify if the reversal of stress fibre formation is due to a reduction in actin polymerization induced by PGI_2_, platelets were allowed to spread on fibrinogen before stimulation with PGI_2_ (10 nM) for 10 minutes. The platelets were then lysed and analysed for the content of filamentous actin. Control platelets underwent a 2.0 ± 0.2 fold increase in F-actin polymerisation, which was unaffected by stimulation with PGI_2_ (2.2 ± 0.3 fold increase), suggesting a targeted modulation of stress fibre formation (Fig. 2f).

To confirm the role for cAMP signalling we used forskolin (Fsk) as a direct activator of adenylyl cyclase. The addition of Fsk (1 μM) to adherent platelets led to a significant and sustained reversal of stress fibre formation (Supplementary Figure S4a,b), increased actin nodule formation (Supplementary Figure S4a,c), a reduction in platelet surface area (Supplementary Figure S4a,e), but did not influence actin polymerisation (Fig. 2f) or platelet adhesion (Supplementary Figure S4d). The effects of Fsk were observed at 10 minutes but in contrast to PGI_2_ were maintined for up to 60 minutes (longest time tested) (Supplementary Figure S4).

**Figure 3:**
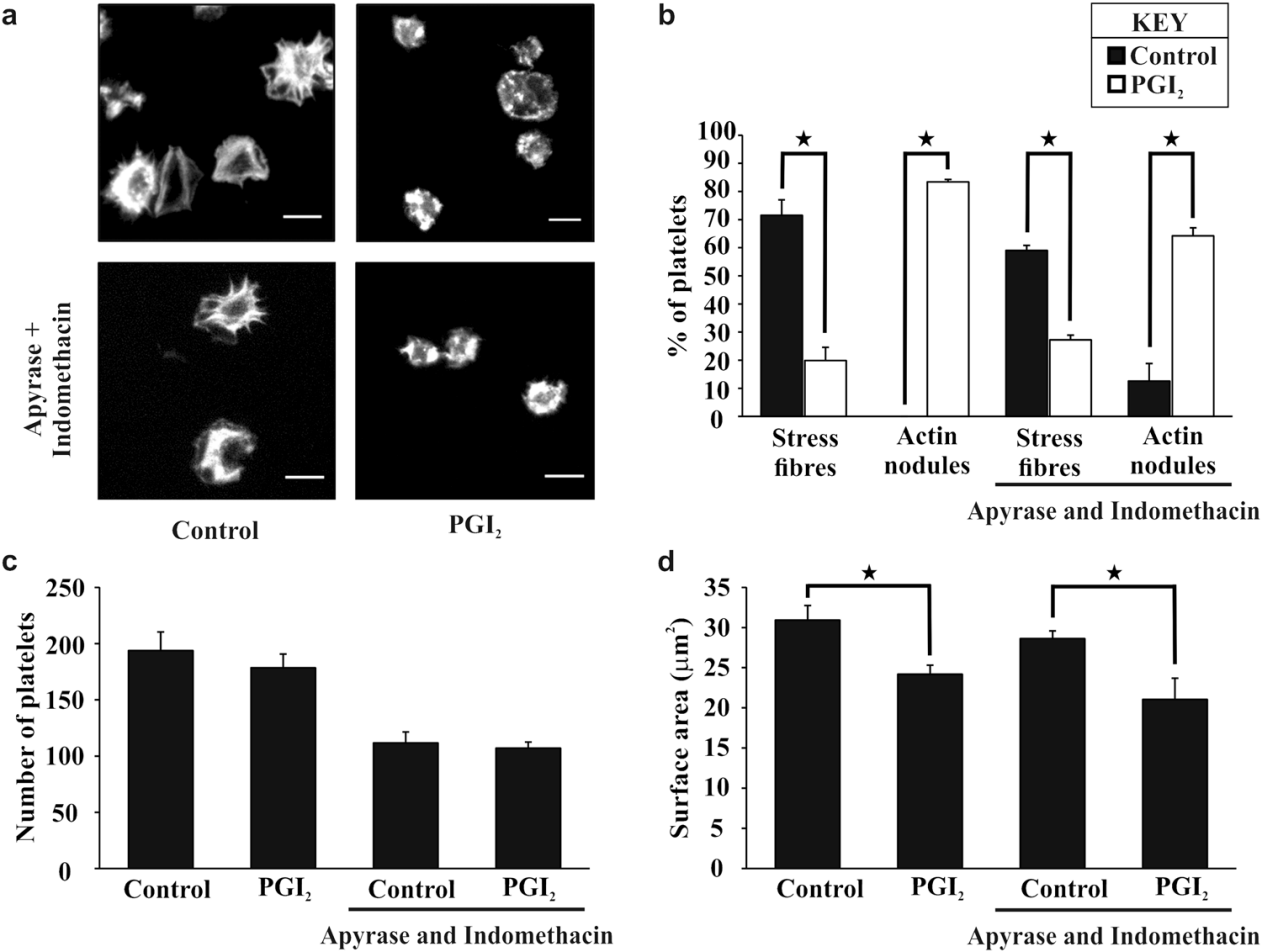
PGI_2_ reverses stress fibre formation independent of ADP and TXA_2_. Platelets (2 × 10^7^/ml) were spread on 100 μg/ml fibrinogen for 25 minutes with or without apyrase (2 U/ml) and indomethacin (10 μM). The platelets were washed, and 10 nM PGI_2_ added for 10 minutes, in the presence or absence of apyrase (2 U/ml) and indomethacin (10 μM). The platelets were then fixed and stained with FITC-phalloidin before being imaged. (**a**) Images are representative of the experimental conditions. (**b**) The number of platelets containing stress fibres or actin nodules in the presence or absence of apyrase (2 U/ml) and indomethacin (10 μM) was identified in control and PGI_2_ treated samples. (**c**) The total number of platelets adhered, was identified in control and PGI_2_ treated samples. (**d**) The average surface area of the spread platelets was identified in control and PGI_2_ treated samples. The experiments are an average of n = 4. Scale bar is 5 μm. p < 0.05.

To determine if the reversal of stress fibres driven by PGI_2_ was independent of effects on secretion, platelets were spread on fibrinogen in the presence of apyrase (2 U/ml) and indomethacin (10 μM) prior to treatment with PGI_2_ (10 nM). The presence of apyrase and indomethacin reduced the number of adherent platelets, but did not influence surface area or actin structures of the adherent platelets. Importantly treatment of adherent platelets with PGI_2_ caused a reversal of stress fibre formation, production of actin nodules, and a reduction in surface area. This demonstrates that the effect of PGI_2_ on the actin cytoskeletal rearrangement is independent of ADP and TXA_2_ bioavailability (Fig. 3). Therefore PGI_2_ reversed both stress fibre and lamellipodia formation, whilst inducing actin nodule formation, independent of platelet secretion.

### PGI_2_ induces cAMP production and activation of the cAMP/PKA pathway in spread platelets

**Figure 4:**
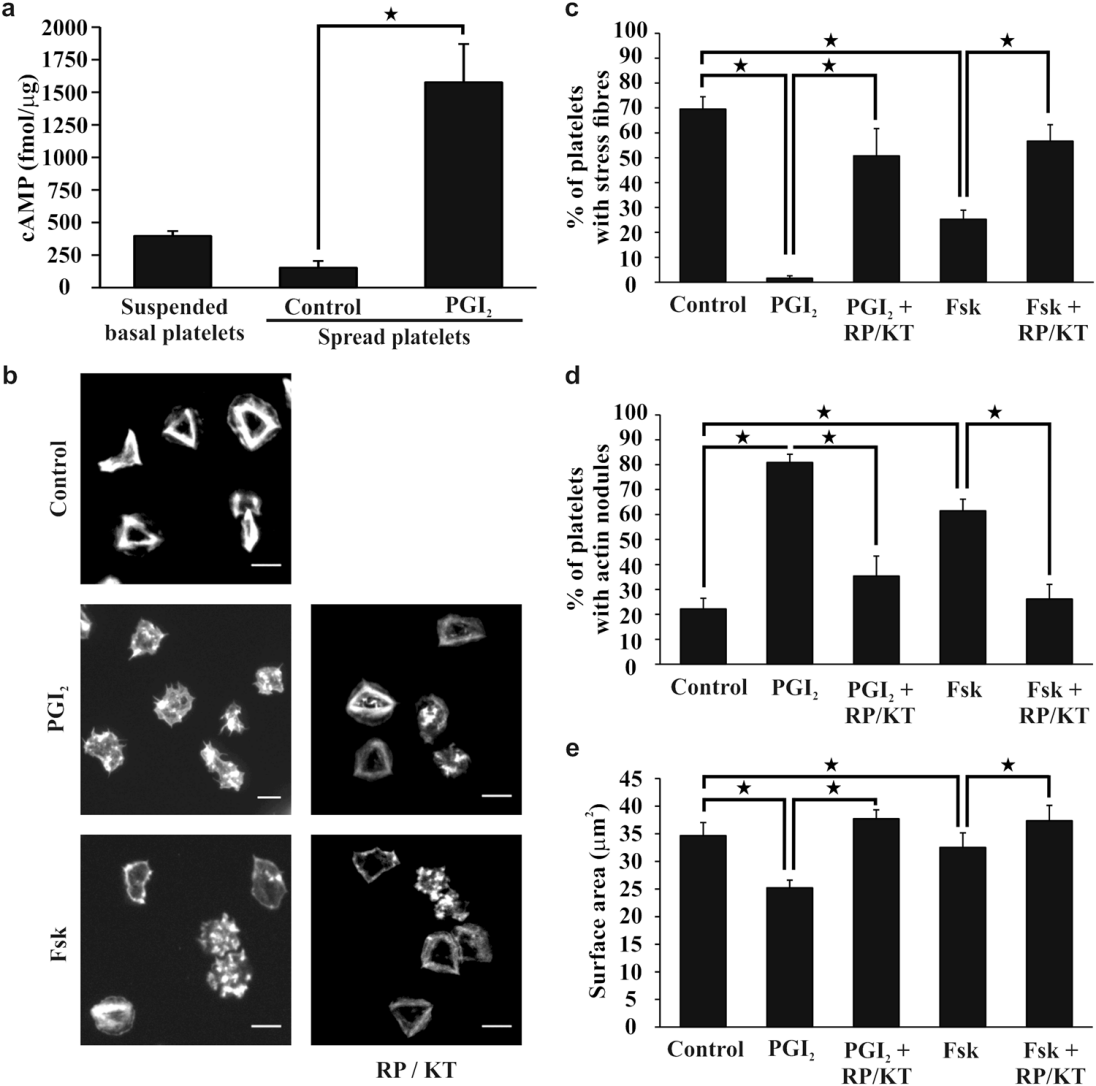
Reversal of platelet stress fibre formation and induction of actin nodule formation is dependent on PKA activation. Platelets (2 × 10^7^/ml) were spread on 100 μg/ml fibrinogen for 25 minutes in the presence or absence of PKA inhibitors; 500 μM RP-8-CPT-cAMP (RP) and 10 μM KT5720 (KT), before being washed with PBS. The platelets were then treated with tyrodes containing 10 nM PGI_2_ or 1 μM forskolin with or without PKA inhibitors 500 μM RP-8-CPT-cAMP and 10 μM KT5720, for a further 10 minutes. The platelets were then fixed, stained with FITC-phalloidin and imaged. (**a**) The levels of cAMP were assessed in basal suspended platelets, control and 10 nM PGI_2_ treated spread platelets. (**b**) Representative images of spread platelets under different experimental conditions. (**c**) The number of spread platelets containing stress fibres were identified for each condition in control and treated samples. (**d**) The number of spread platelets containing actin nodules were identified for each condition in control and treated samples. (**e**) The average surface area of the spread platelets was analysed for each condition in control and treated samples using Image J. The experiments are an average of n = 3. p < 0.05. Scale bar is 5 μm.

PGI_2_ induces generation of cAMP and activation of PKA in platelets, although PKA-independent signalling is shown in other cell types^23, 24^. Initially it was confirmed that adherent platelets could synthesise cAMP. Treatment of adherent platelets with PGI_2_ (10 nM) for 2 minutes led to a significant increase in cAMP from 152 ± 52 fmol/μg to 1576 ± 293 fmol/μg (p < 0.05) and was consistent with platelets in suspension stimulated with PGI_2_ for 1 minute^25^ (Fig. 4a). To confirm that the actin cytoskeletal rearrangements induced by PGI_2_ were mediated by PKA the established PKA inhibitors Rp-8cpt-cAMPs (500 μM) and KT5720 (10 μM) were employed^26, 27^. The presence of Rp-8cpt-cAMPs (500 μM) and KT5720 (10 μM) blocked the ability of PGI_2_ to reverse stress fibres, induce actin nodule formation, and reduce platelet spreading (Fig. 4b–e). This data suggests PGI_2_ modulates the actin cytoskeleton in activated spread platelets via a cAMP/PKA dependent mechanism.

**Figure 5:**
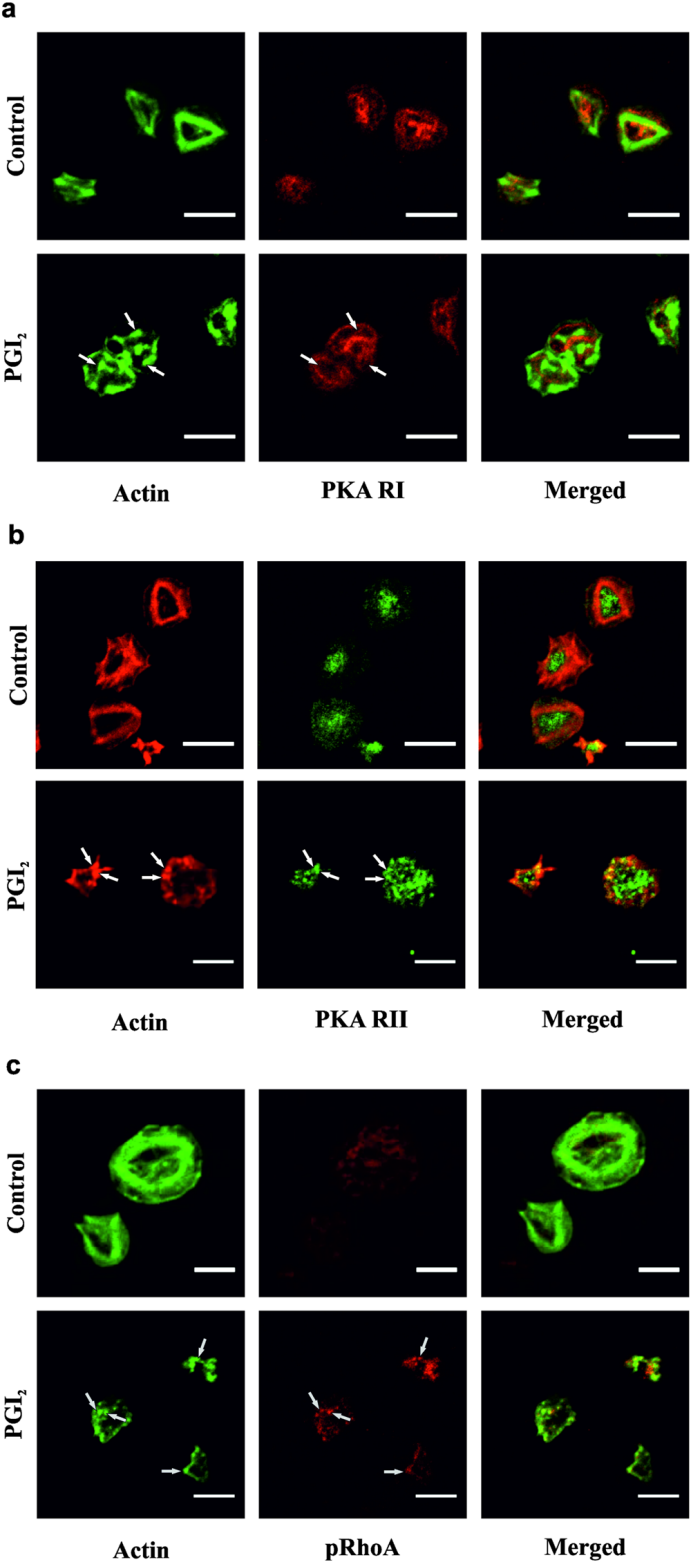
Actin nodules contain PKA signalling proteins. Platelets (2 × 10^7^/ml) were spread on 100 μg/ml fibrinogen for 25 minutes before being washed with PBS. The platelets were then treated with tyrodes with or without 10 nM PGI_2_ for a further 10 minutes. The platelets were then fixed, lysed and stained for either (**a**) PKA RI (1:100), (**b**) PKA RII (1:100) or (**c**) pRhoA (1:1000); and co-stained with actin (FITC-Phalloidin) for 60 minutes, before mounting and imaging the slides. Images are representative of at least 3 experiments. Scale bar is 5 μm.

Actin nodules represent a pre-stress fibre actin structure^9^ and our observation that cAMP signalling was associated with the reversal of stress fibres led us to examine these nodules in more detail. Firstly, adherent platelets were stained for PKARI and RII subunits. Consistent with our previous observations, differential localisation of these isoforms was observed with PKARI found at the periphery of the platelet and PKARII distibuted throughout the platelet (Fig. 5a,b)^28^. Moreover, PKARII was localized with the actin nodule, while PKARI staining was mutually exclusive to the actin cytoskeleton. PKA activity is localised to particular cell compartments in order to focus its catalytic activity on to specific substrates. Further analysis using immunofluorescence demonstrated the presence of phosphorylated PKA substrates including pRhoA^ser188^ at actin nodules present in spread platelets after being treated with PGI_2_ (Fig. 5c). This suggested that cAMP maybe focussed on the actin nodule associated RhoA.

### PGI_2_ reverses activation of RhoA in spread platelets

**Figure 6:**
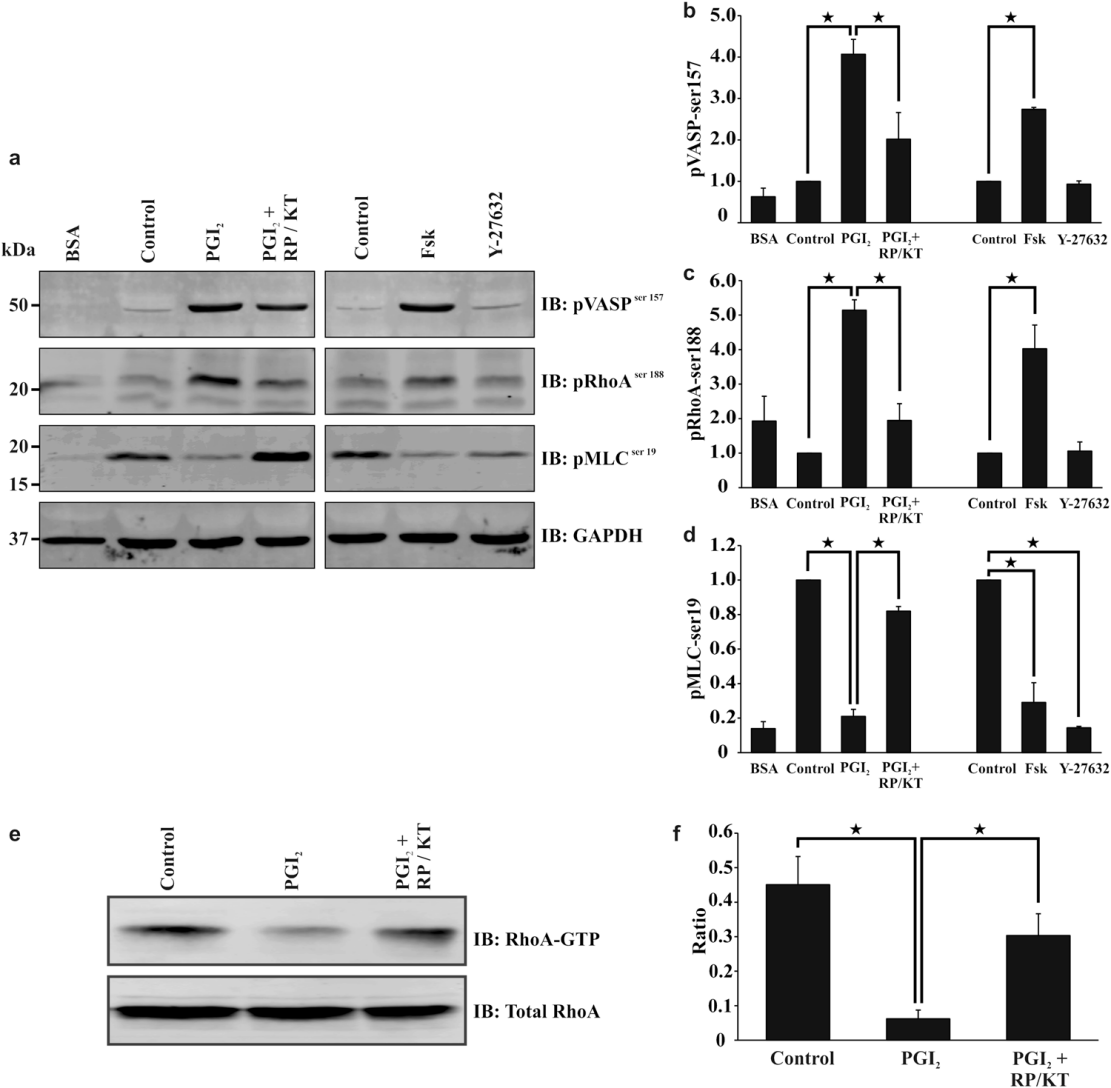
PGI_2_ induces a PKA signalling response in spread platelets. Platelets (2 × 10^8^/ml) were spread on 100 μg/ml fibrinogen for 25 minutes in the presence or absence of PKA inhibitors 100 μM RP-8CPT-cAMP (RP) and 2 μM KT5720 (KT), before being washed with PBS. (**a**) The platelets were then treated with tyrodes containing 10 nM PGI_2_ with or without PKA inhibitors (100 μM RP-8CPT-cAMP and 2 μM KT5720), or 1 μM forskolin, or Y27632 (10 μM), for a further 10 minutes. The samples were then lysed with laemelli buffer before being western blotted for pVASP^ser159^, pMLC^ser19^, pRhoA^ser188^, and GAPDH. Cropped gel Images are representative of at least three experiments (full length gels are illustrated in Supplementary Figure S6). (**b**–**d**) Densitometry for the western blots; pVASP^ser159^, pMLC^ser19^ and pRhoA^ser188^ using GAPDH as the loading control. The ratios were standardised to the control. (**e**) Spreading the platelets as above, they were treated with tyrodes containing 10 nM PGI_2_ with or without PKA inhibitors 100 μM RP-8CPT-cAMP and 2 μM KT5720 for a further 10 minutes. The samples were then lysed, before the addition of RhoA GTP beads. Samples were then western blotted for active RhoA and total RhoA. Cropped gel Images are representative of at least three experiments (full length gels are illustrated in Supplementary Figure S6). (**f**) Images of RhoA pull down were analysed for densitometry (full length gels are illustrated in Supplementary Figure S6). Analysis is an average of at least n = 3 experiments. p < 0.05.

Platelet stress fibre formation requires the activation of RhoA and its downstream effector ROCK^9^. Platelets incubated with the ROCK inhibitor Y27632 or the RhoA inhibitor Rhosin prior to spreading fail to form stress fibres (Supplementary Figure S5)^8^. As Fig. 2 had identified a significant reversal of stress fibre formation in the presence of PGI_2_, this indicated that PGI_2_ could reverse RhoA activation. To examine this possibility, platelets were spread for 25 minutes, treated with PGI_2_ (10 nM) or Fsk (1 μM) and the phosphorylation of key proteins evaluated. Confirmation of cAMP signalling in adherent platelets was illustrated by the sustained VASP-ser^157^ phosphorylation in response to PGI_2_ or Fsk (Fig. 6a,b, Supplementary Figure S6 and data not shown). In untreated adherent platelets RhoA was non-phosphorylated. In contrast phosphoRhoA-ser^188^ became apparent after PGI_2_ (10 nM) and Fsk (1 μM) stimulation, in a PKA dependent manner (Fig. 6a,c, Supplemetary Figure S6 and data not shown). Phosphorylation of RhoA is associated with RhoA inactivation, as it binds the GDI subunit, and therefore can no longer cause the activation of ROCK^29^. Analysis of RhoA showed it was activated (GTP-bound) in adherent platelets, but that addition of PGI_2_ significantly reduced the active RhoA present within the spread platelets (Fig. 6e,f and Supplementary Figure S6). Further to this, and in agreement with a reduction in RhoA activity, PGI_2_ and Fsk reduced MLC-ser^19^ phosphorylation in spread platelets (Fig. 6a,d and Supplementary Figure S6). These results indicate that PGI_2_ can inhibit the activated RhoA via phosphorylation by a PKA-dependent mechanism.

## Discussion

Haemostasis relies on stable platelet adhesion for subsquent thrombus formation. The ability of platelets to undergo a rapid remodelling of the actin cytoskeleton is critical in order to withstand shear stress. Indeed an inability to form lamellipodia or stress fibres upon platelet activation and spreading is linked to thrombus instability and embolisation^8, 12, 13^. However, under physiological conditions these key changes occur in situations where platelets are continually exposed to PGI_2_. Given the key role of RhoGTPases in actin remodelling we examined the influence of cAMP signalling on activated platelets, using the platelet spreading and *in vitro* flow assays. We show here that (i) PGI_2_ can cause the reversal of surface area of platelet aggregates under high shear (ii) PGI_2_ can reverse platelet spreading via dissolution of stress fibres, and the formation of actin nodules in a cAMP/PKA dependent mechanism, (iii) PKA localises to the actin nodule, using it as a platform to mediate PKA mediated inhibitory phosporylation of RhoA. These data suggest that in addition to its already known roles in causing platelet inhibition prior to thrombus formation, cAMP signalling can control the size and stability of existing platelet aggregates via modulating the actin cytoskeleton, causing the aggregates to become vulnerable to high shear stress.

PGI_2_ and the PKA signaling pathway have been implicated in actin cytoskeleton modulation in multiple cell types^15, 20, 21^. Although prior incubation of platelets with PGI_2_ and Fsk leads to inhibition of platelet adhesion, spreading and thrombus formation^30–33^, it is unclear if PGI_2_ can actively modulate a fully activated platelet. This is a critical idea as it demonstrates that PGI_2_ plays a dual role in the control of thrombus formation, both inhibition of platelets prior to activation, and reversal of platelets once they are already active. Both effects could then lead to reduction in thrombus formation. The reversal of platelet activation links into recent modelling of thrombus formation that suggests a dynamic structure with a core area, within which the platelets are fully activated and surrounded by a pheripheral area in which the activatory signals are much weaker^2^. The balance between the activatory and inhibitory signals acting on the platelets likely determines their activatory status. Therefore we set out to explore how PGI_2_ affected preexisting platelet aggregates. Using an *in vitro* flow assay we show for the first time that post perfusion of thrombi on fibrinogen with PGI_2_ caused a significant reduction in surface area. The ability of the platelet to withstand high shear is driven, at least in part, by the formation of remodelled actin structures within the platelet and we focussed our investigation on these processes. Platelet spreading was associated with progressive changes in actin structures including filopodia, actin nodules, lamellipodia and ultimately stress fibres. Addition of PGI_2_ to spread platelets induced cytoskeletal remodelling associated with stress fibre dissolution consistent with previous observations in other cell types^22, 34^. The effects of PGI_2_ were mimicked by forskolin, but blocked by PKA inhibitors, indicating a central role for the cAMP-PKA signalling cascade.

To examine the mechanism that underpinned stress fibre disassembly and actin nodule formation we used a pharmacolgical approach. Incubation of platelets with RhoA and ROCK inhibitors, led to adherent platelets rich in actin nodules and an absence of stress fibres as reported previously^8^. Since the addition of PGI_2_ led to a loss of stress fibres, we speculated that PGI_2_ may target RhoA signalling in adherent platelets. Consistent with our previous studies in suspended platelets we found that PGI_2_ increased the inhibitory phosphorylation of RhoA at ser^188^ and reduced the GTP-loading of RhoA. Here we have extended our original findings to show that cAMP signalling can switch off activated RhoA in addition to blocking its activation. The inhibition of RhoA/ROCK signalling led to dephosphorylation of MLC, which likely occurs through the ability of cAMP signalling to induce disinhibition of MLCP. Moreover we show that the inhibitory phosphorylation of RhoA identified by immunoblotting is primarily located at the actin nodule. The actin nodule was identified as an F-actin rich structure that was formed in spreading platelets prior to lamellipodia formation^9^ that required both the Arp2/3 complex and WASp, and are sites of tyrosine kinase signalling activity^35^. Given that RhoA phosphorylation is mediated by PKA we also examined the presence of the kinase. cAMP signalling events are highly regulated and involve the selective coupling of cAMP signalling complexes to specific substrates or regions within the cell (Reviewed in ref. 36). Our data suggest that the actin nodule may act as such a signalling platform allowing localisation of PKAII, but not PKAI, and several phosphorylated PKA substrates. These data suggest that PKAII specifically targets RhoA and that this inhibitory phosphorylation prevents sustained stress fibre formation allowing reversion to actin nodules. This is in addition to the previous identification of Rac puts forward the interesting hypothesis that the nodule is under the control of both Rac and RhoA, and that the interplay between the level of activation of both is critical to actin nodule formation and dissolution.

This reversal of stress fibres on fibrinogen links with the thrombus defect as stress fibre formation is critical to withstand high shear^8^. The physiogical importance of our observations relates to the environment of the growing thrombus. The data indicates that the reversal of platelet aggregate formation by PGI_2_ is due to its effect on the upper layers of the thrombus, which are linked by the exposure of fibrinogen. Furthermore, thrombin and TXA_2_ generated at the sites of thrombus formation further activate platelets but also stimulate the biosynthesis of PGI_2_ by the blood vessel wall^4^. It is possible that in addition to preventing excessive platelet activation the release of PGI_2_ in areas of vascular damage could modulate thrombus formation through regulating thrombus stability. Indeed the model proposed by Welsh *et al*.^3^ of graded platelet activation through the thrombus could be explained at least in part by our observations. Thrombi have been postulated to be made up of a core area, within which the platelets are fully activated as they have a multitude of activatory signals. This core area is surrounded by a peripheral area where the activatory signals are much weaker, and platelets are known to bind transiently to the growing thrombi. Exposure of these peripheral platelets to PGI_2_ may facilitate their embolisation and contribute to the control of thrombosis. It would be highly interesting to extend this work through understanding how this effect of platelet reversal interplays with the activation of the coagulation system, and the formation of fibrin, through the action of thrombin. Thrombin activation of platelets has previously been identified to be inhibited by PGI_2_, so can PGI_2_ reverse its activation? How would this reversal affect the formation of fibrin? Indeed, is PGI_2_ reversal of platelet activation required to prevent excessive fibrinogen exposure, and therefore excessive fibrin formation. This potentially ties in with the altered fibrin formation associated with thrombi from patients with pulmonary arterial hypertension. Within this condition there is reduced PGI_2_ production, and thrombi with altered fibrin formation, that is resistant to lysis^37^. Therefore is the reduction in PGI_2_ inducing excessive fibrin formation due to an inability to reverse platelet activation? Furthermore given the importance of PGI_2_ in both the initial inhibition, and also the reversal of thrombus formation, this data strengthens the case for the development of anti-platelet drug therapies that target cAMP elevation for the control of thrombus formation.

However, importantly this report identifies PGI_2_ mediated reversal of platelet activation. This reversal of activation, through the control of activity of RhoA, could have a signficant effect upon the capability of the platelet to work effectively in a high shear environment. This report therefore demonstrates a novel mechanism by which PGI_2_ regulates platelet activation through continous modulation of RhoA and stress fibre formation.

## Material and Methods

### Materials

PGI_2_ (Cayman Chemical, Michigan, USA), Forskolin (Sigma-Aldrich, UK), Fibrinogen (Enzyme Research, Swansea, UK), Y-27632 (Abcam, Cambridge, UK), Rp-8CPT-cAMP (Biolog, Bremen, Germany), KT5720 (Abcam, Cambridge, UK), pRhoA^ser188^ (Santa-Cruz Biotechnology, Heidelberg, Germany), pMLC^ser19^ and pVASP^ser157^ (New England Biolabs, Hitchin, UK), GAPDH and Arp2/3 (Millipore, Watford, Hertfordshire, UK), PKA RI (Cell Signalling Technology, Leiden, Netherlands), PKARII (BD Biosciences, Oxford, UK), pPKA substrate (Cell Signalling Technology, Leiden, Netherlands), WASP (Santa-Cruz Biotechnology, Heidelberg, Germany), pTyrosine (Cytoskeleton, Denver, UK), RhoA pulldown kit (Cytoskeleton, Denver, UK), cAMP assay and ProLong Diamond Antifade Mountant (GE healthcare, Little Chalfont, Buckinghamshire, UK), Flourescent secondary anti-mouse 800 and anti-rabbit 680 antibodies (LI-COR Biotechnology, Cambridge, UK) All other chemicals were from Sigma Ltd (Poole, UK) unless otherwise stated.

### Platelet preparation

Whole blood was mixed with acid-citrate dextrose (ACD; 5:1) (114 mM glucose, 30 mM Tris-Na Citrate, 72.6 mM NaCl and 3.0 mM citric acid pH 6.4) and platelets were isolated using low pH as previously described^18^. Platelets were allowed to rest for 30 minutes prior to experimentation. Written informed consent was acquired for the donation of blood. The work was conducted in accordance with the relevant guidelines and regulations and was completed under the ethical permission granted by the Hull York Medical School ethical committee, for “The study of platelet activation, signalling and metabolism”.

### Platelet Spreading

Coverslips were incubated with fibrinogen (100 μg/ml) overnight at 4 °C, washed with PBS and blocked with denatured fatty acid free BSA (5 mg/ml) for 1 hour at room temperature. Platelets (2 × 10^7^/ml) were adhered to immobilised proteins for 25 minutes at 37 °C in the presence or absence of Apyrase (2 U/ml), Indomethacin (10 μM), or Rp-8-CPT-cAMP (500 μM) and KT5720 (10 μM). After 25 minutes the coverslips were washed twice with PBS to remove nonadhered platelets and then treated with PGI_2_ (1-1000 nM) or forskolin (0.1–100 μM) in the presence or absence of Apyrase (2 U/ml), Indomethacin (10 μM), or Rp-8-CPT-cAMP (500 μM) and KT5720 (10 μM). At the required timepoint the platelets were fixed with 4% paraformaldehyde for 10 minutes, lysed with 0.1% Triton X-100, stained with FITC-phalloidin and if required the relevant primary antibody (PKARI, and PKARII (1:100), pRhoA^ser188^, Arp2/3, and 4G10 (1:1000)), followed by the secondary antibody (1:200), mounted and visualized using a Zeiss Axio Observer (Zeiss, Cambridge, UK) with a x63 oil immersion objective (1.4 NA) and Zen Pro software (Carl Zeiss, Cambridge, UK). Images of spread platelets were analysed using ImageJ software (NIH, Bethesda, USA) to identify the surface area, the platelets containing either actin nodules or stress fibres, and platelet adhesion. In some cases platelets (2 × 10^7^/ml) were preincubated with PGI_2_ (1–1000 nM) or Forskolin (0.1–100 μM), for 2 minutes prior to adhesion for 45 minutes.

### *In vitro* Flow Assay

Flow studies were performed with multichannel biochips (Cellix, Dublin Ireland). Biochips were coated with 300  μg/ml fibrinogen overnight at 4 °C and blocked with denatured BSA (5 mg/ml) for 1 hour. Whole blood was stained with DIOC_6_ (10 μM) and flowed through the biochips for 2 minutes at a shear rate of 1000 s^−1^ at 37 °C. Biochips then underwent a post-flow with Tyrode’s buffer supplemented with or without PGI_2_ (100 nM; 1000 s^−1^ at 37 °C) for 10 minutes, before fixation with 4% paraformaldehyde, staining overnight with DiOC_6_ (10 μM) and imaging using a Apotome.2 confocal unit on a Zeiss Axio Observer (Zeiss, Cambridge, UK) with a x63 oil immersion objective (1.4 NA) and Zen Pro software (Carl Zeiss, Cambridge, UK). Analysis of the surface area coverage of each condition was performed using ImageJ software (NIH, Bethesda, USA).

### Immunoblotting

Six well plates were coated with fibrinogen as per platelet spreading. Platelets (2 × 10^8^/ml) were adhered for 25 minutes before removal of non-adherent platelets. Adherent platelets were then treated with PGI_2_ (10 nM), FSK (1 μM), or Y27632 (10 μM) in the presence or absence of Rp-8CPT-cAMP (500 μM) and KT5720 (10 μM). Platelets were lysed with Laemelli buffer and the lysates were separated by SDS-PAGE, transferred and immunoblotted with the required antibodies (pVASP^ser157^ (1:1000), pRhoA^ser188^ (1:1000), pMLC^ser19^ (1:1000), or GAPDH (1:6000)). The blots were imaged by Odyssey CLx Infrared Imaging system (LI-COR Biotechnology, Cambridge, UK), and densitometry was analysed by Image Studio ver.5.2 (LI-COR Biotechnology, Cambridge, UK).

### F-actin Analysis

Six well plates were coated with fibrinogen as per platelet spreading. Platelets (2 × 10^8^/ml) platelets were adhered for 25 minutes before being washed with PBS. The platelets were then treated with Tyrode’s buffer, PGI_2_ (10 nM) or FSK (1 μM) for 10 minutes. The samples were processed as previously described^38^. Aliquots of unstimulated platelets (1 × 10^8^/ml to 8 × 10^8^/ml) were used to calculate both the protein concentration and F-actin concentration at these platelet numbers as an unstimulated control.

### Measurement of cAMP and RhoA Pull-down assay

Six well plates were coated with fibrinogen as per platelet spreading. Platelets (2 × 10^8^/ml) were spread for 25 minutes before washing, and treatment with Tyrodes buffer or PGI_2_ (10 nM) for 2 minutes. Platelets were lysed and cAMP concentrations measured using a commerical available assay kit^18^. For the RhoA pull down platelets the samples were prepared as above, but then lysed and lysates incubated for 90 minutes at 4 °C with Rhotekin-RBD-beads. Bead pellets were washed once and Laemmeli buffer was added prior to immunoblotting as previously published^39^.

### Statistical analysis

Results are shown as mean ± standard error of mean (SEM). Data was arcsin transformed as appropriate and then subject to statistical analysis. Statistical analysis was performed by using one-way ANOVA with a P value of < 0.05.

## Electronic supplementary material

Supplementary Figure

Supplementary Video 1

Supplementary Video 2

Supplementary Video 3
