# Supplementary material for: Prostacyclin reverses platelet stress fibre formation causing platelet aggregate instability
Source: Sci Rep. 2017 Jul 17;7:5582. doi: 10.1038/s41598-017-05817-9 (PMC5514131; doi:10.1038/s41598-017-05817-9)
Supplement: Supplementary file 1 — Supplementary Figure [file 41598_2017_5817_MOESM1_ESM.pdf]

Prostacyclin reverses platelet stress fibre formation causing platelet aggregate instability

Authors:
MZ Yusuf, Z Raslan, L Atkinson, A Aburima, SG Thomas, KM Naseem and *SDJ Calaminus.

Supplementary Information

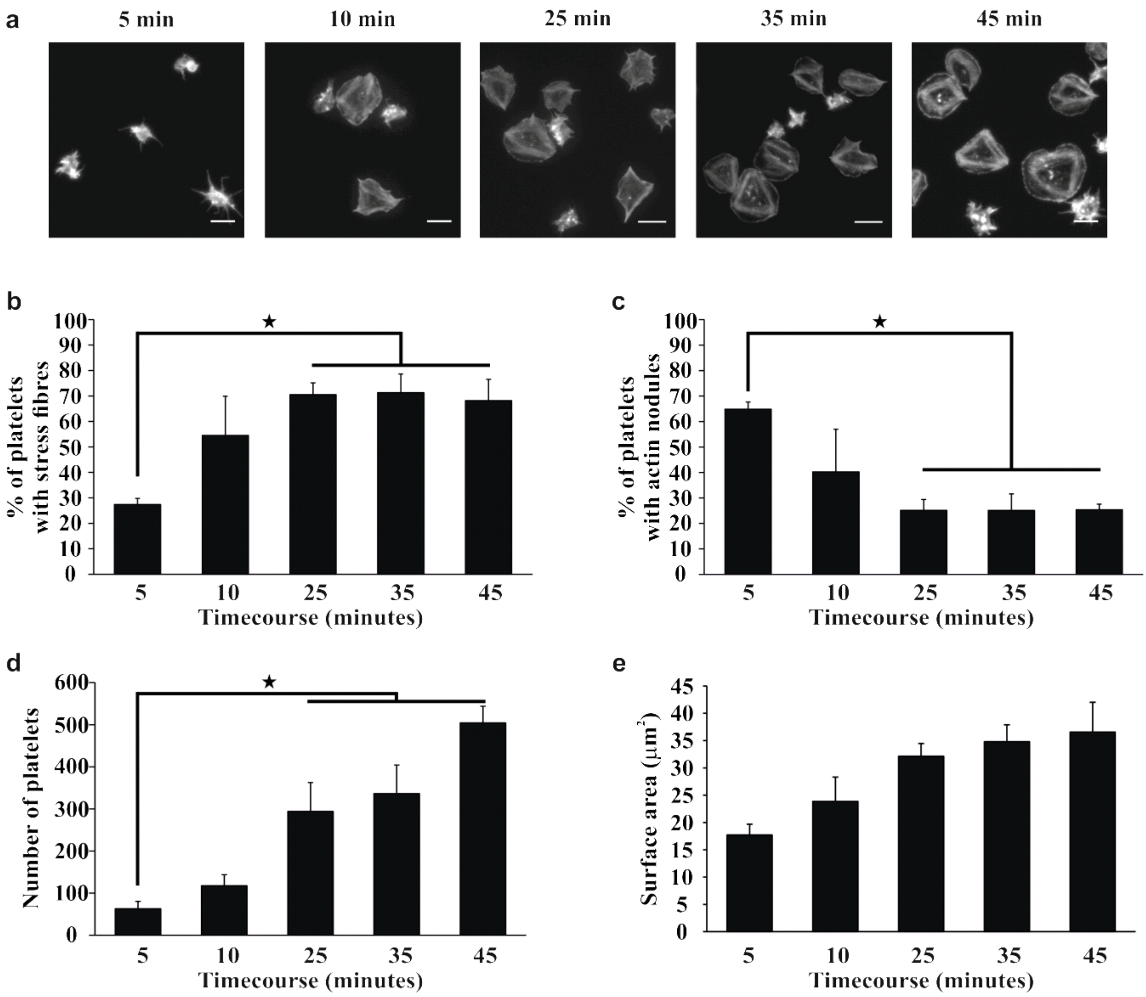

Supplementary Figure S1: Spreading profile of platelets. Platelets ($2x10^7$/ml) were spread on 100μg/ml fibrinogen for a duration of 5 - 45 minutes. After their respective time points of spreading, the platelets were washed with PBS and were then fixed. These fixed platelets were then stained with FITC-phalloidin before being imaged. **a)** Images are representative of the experimental conditions. **b)** The number of platelets containing stress fibres at different time points of spreading. **c)** The number of platelets containing actin nodules at different time points of spreading. **d)** The total number of platelets adhered for these time points. **e)** The average surface area of the spread platelets was identified for these time points. The experiments are an average of n=3. Scale bar is 5μm. $p<0.05$.

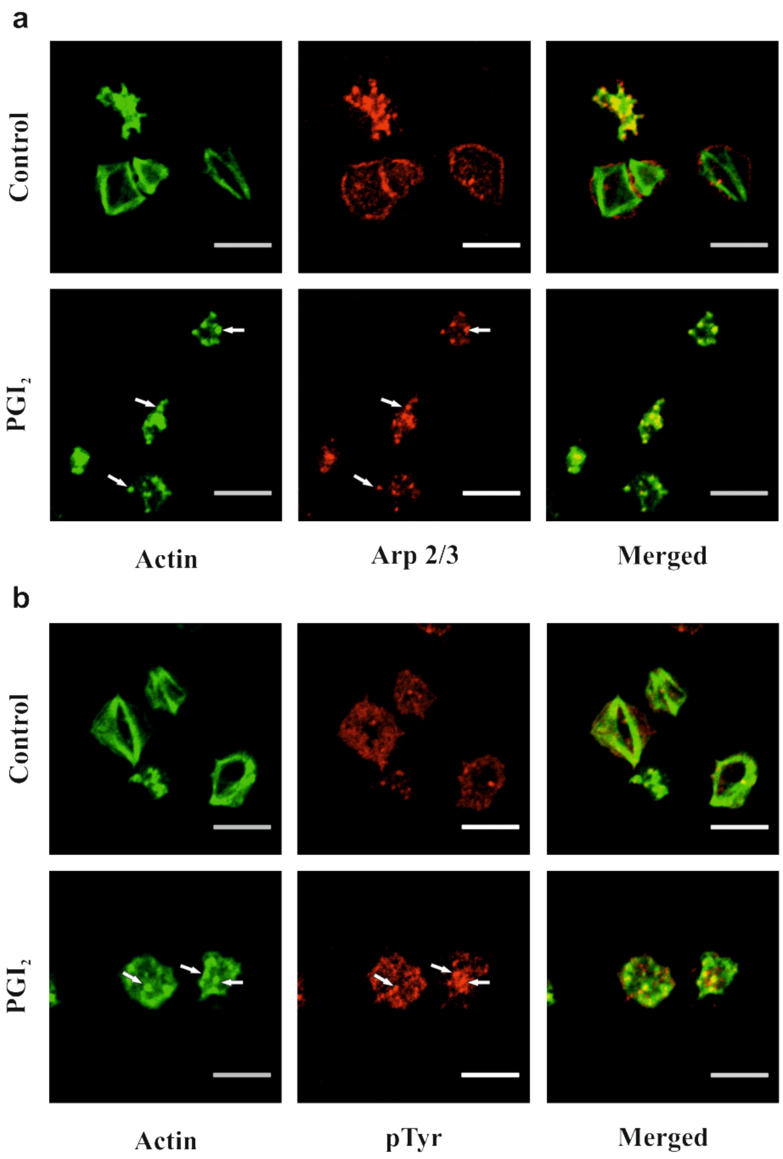

Supplementary Figure S2: Confirmation of the presence of Actin nodules. Platelets ($2x10^7$/ml) were spread on 100µg/ml fibrinogen for 25 minutes before being washed with PBS. The platelets were then treated with tyrodes with or without 10nM PGI_2 for a further 10 minutes. The platelets were then fixed, lysed and stained for either **a)** Arp2/3 (1:1000) or **b)** pTyr (1:1000); and co-stained with actin (FITC-Phalloidin) for 60 minutes, before mounting and imaging the slides. Images are representative of at least 3 experiments. Scale bar is 5µm.

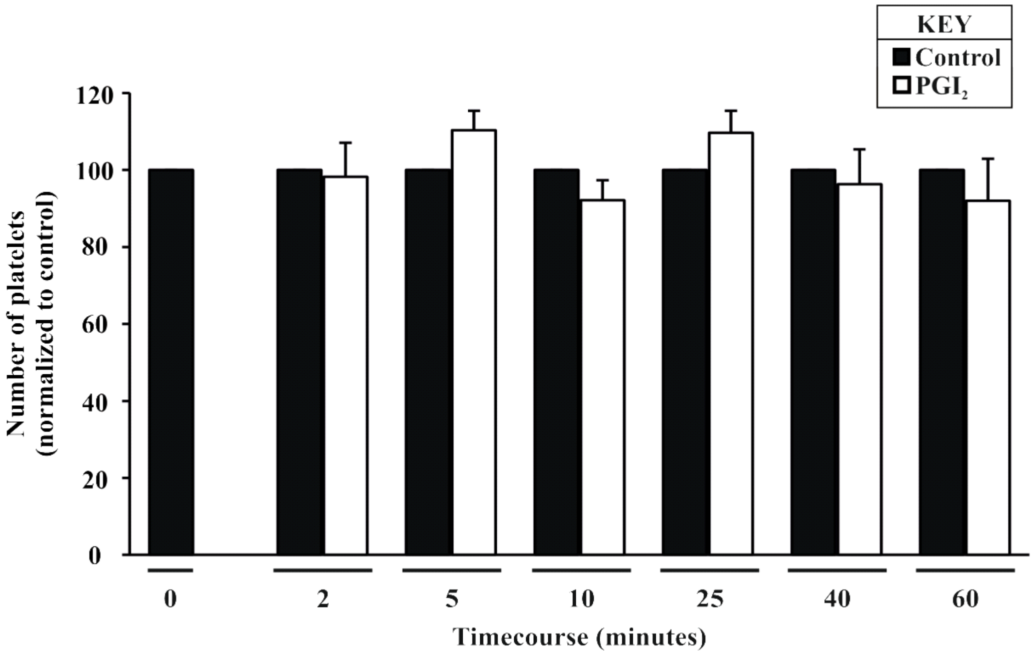

Supplementary Figure S3: Adhesion of spread platelets upon exposure to PGI_2 is not affected. Platelets ($2x10^7$/ml) were spread on 100µg/ml fibrinogen for 25 minutes before being washed with PBS. The spread platelets were treated with PGI_2 for 10 minutes. The platelets were then fixed, stained with FITC-phalloidin and visualised under a microscope. The experiments are an average of n=3.

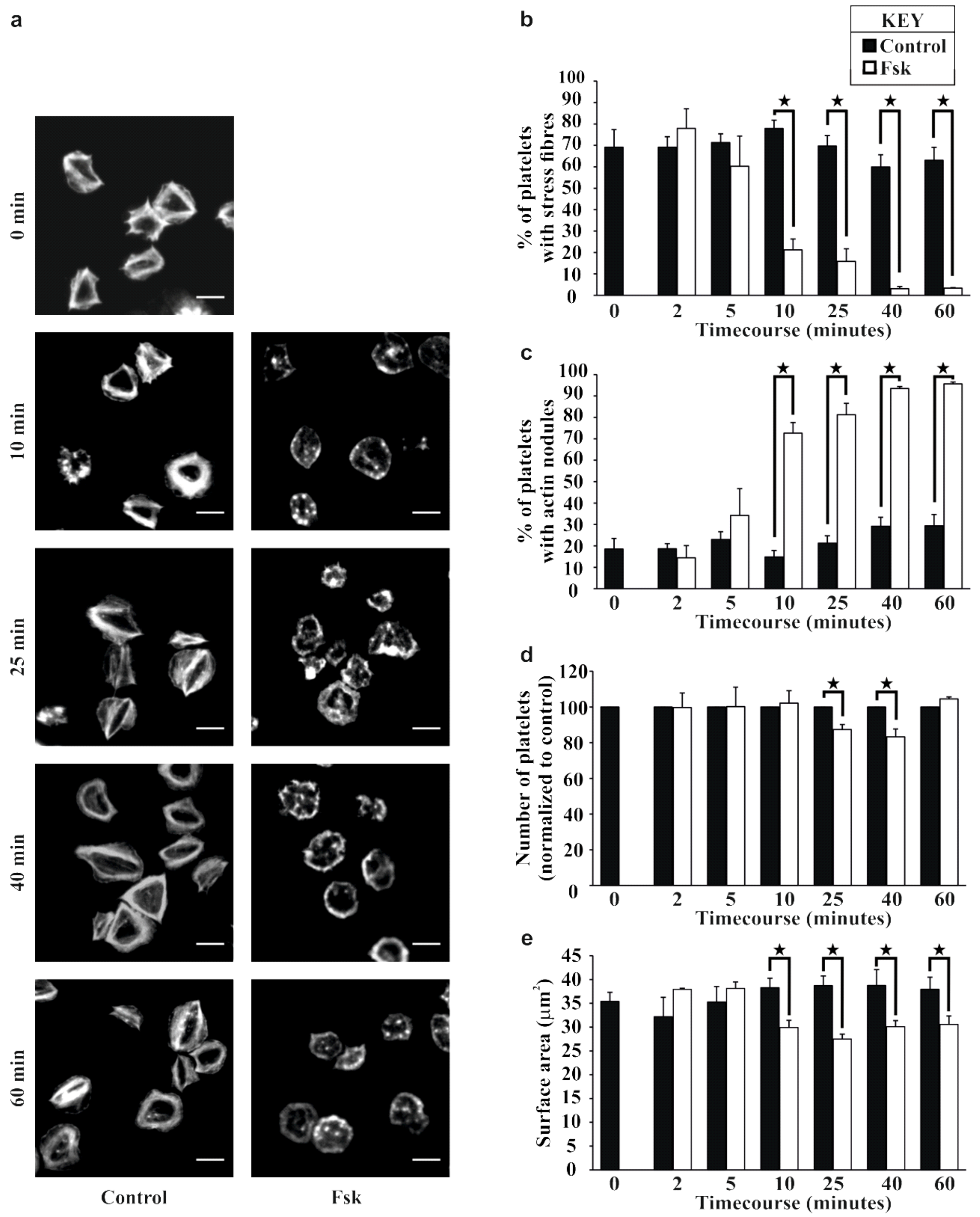

Supplementary Figure S4: Post treatment of Forskolin (Fsk) induces stress fibre reversal in platelets spread on fibrinogen in a time dependent manner. Platelets ($2x10^7$/ml) were spread on 100μg/ml fibrinogen for 25 minutes, before being washed with PBS, and then 1μM forskolin was added, for a further 2-60 minutes. The platelets were then fixed and stained with FITC-phalloidin before being imaged via fluorescent microscopy. **a)** Representative images of each condition of the experiment. **b)** The number of spread platelets containing stress fibres was identified in control and Fsk treated samples. **c)** The number of spread platelets containing actin nodules was identified in control and Fsk treated samples. **d)** The adhesion of platelets was analysed for each timepoint in control and Fsk treated samples. **e)** The average surface area of the spread platelets was analysed for each timepoint in control and Fsk treated samples using Image J. The experiments are an average of n=3. $p<0.05$. Scale-bar 5μm.

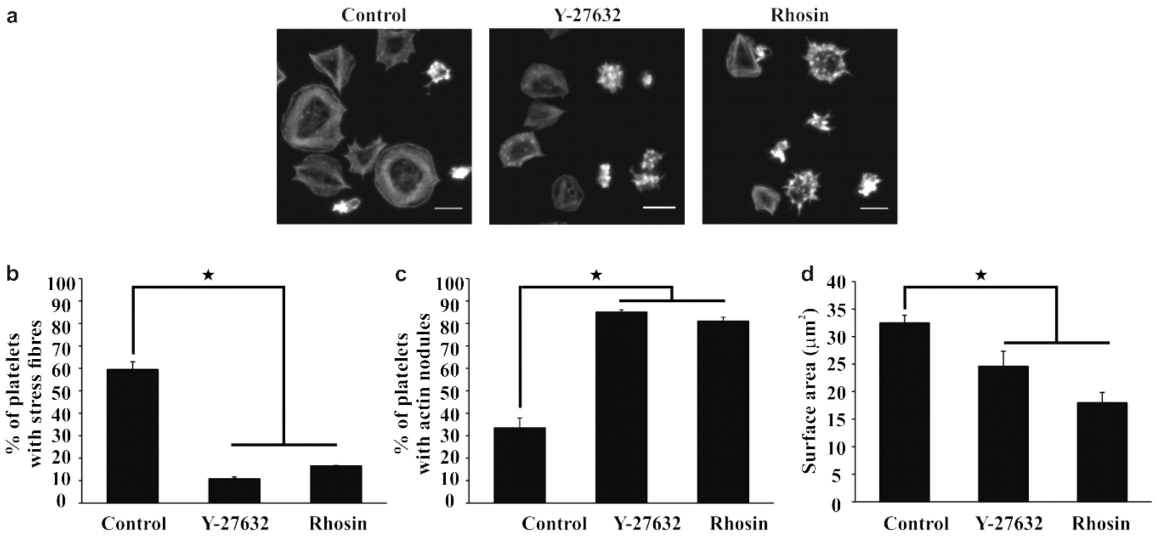

Supplementary Figure S5: A reduction in stress fibres with a reciprocal increase in actin nodules by treating platelets with Y27632 and/or Rhosin prior to spreading them on fibrinogen. Platelets ($2x10^7$/ml) were treated with RhoA-ROCK pathway inhibitors (10μM Y27632 or 10μM Rhosin) prior to spreading them on 100μg/ml fibrinogen for 45 minutes. The platelets were then fixed and stained with FITC-phalloidin before being imaged. **a)** Images are representative of three experiments. **b)** The number of platelets containing stress fibres whilst their treatment with the inhibitors. **c)** The number of platelets containing actin nodules whilst their treatment with the inhibitors. **d)** The average surface area of the spread platelets after their treatment with the inhibitors. The experiments are an average of n=3. Scale bar is 5μm. $p<0.05$.

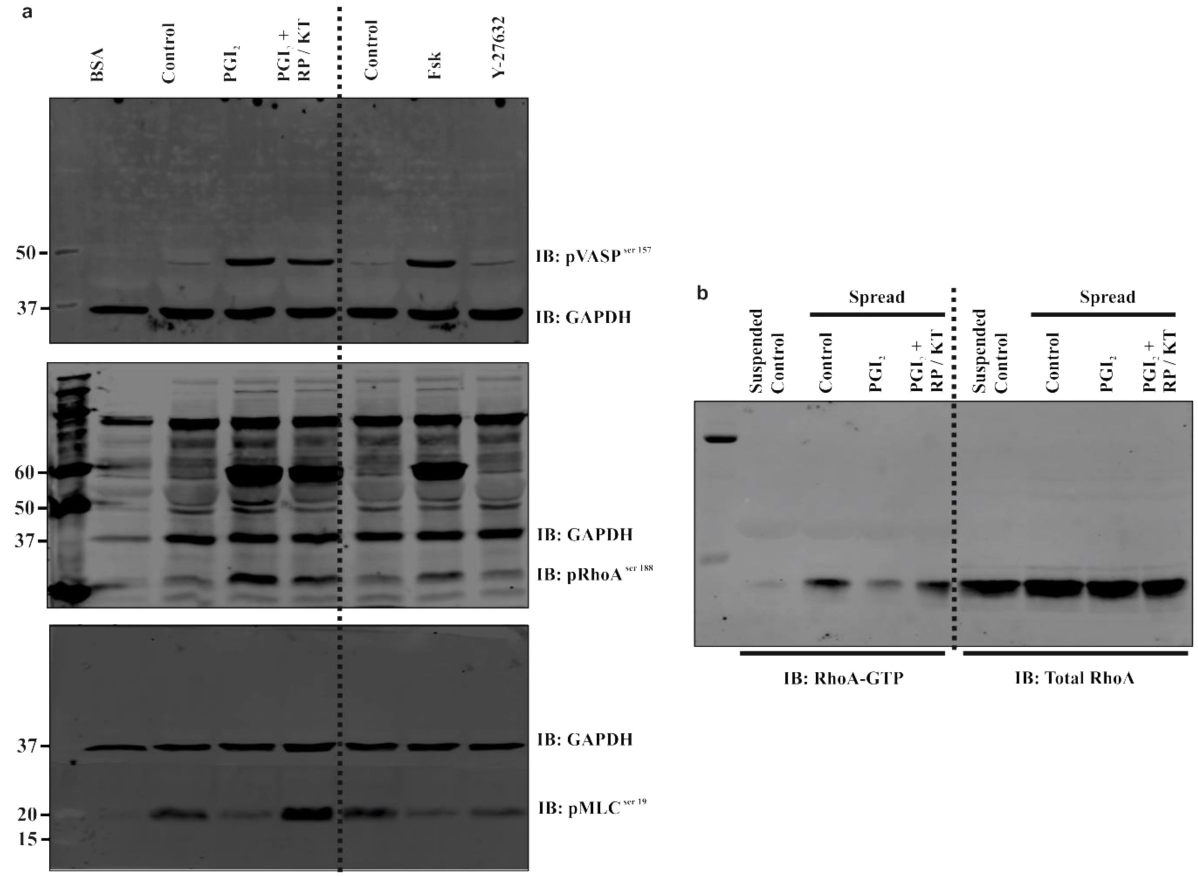

Supplementary Figure S6: Representative complete blots for PGI_2 induced PKA signalling response in spread platelets. Platelets ($2x10^8$/ml) were spread on 100μg/ml fibrinogen for 25 minutes in the presence or absence of PKA inhibitors 100μM RP-8CPT-cAMP (RP) and 2μM KT5720 (KT), before being washed with PBS. **a)** The platelets were then treated with tyrodes containing 10nM PGI_2 with or without PKA inhibitors (100μM RP-8CPT-cAMP and 2μM KT5720), or 1μM forskolin, or Y27632 (10μM), for a further 10 minutes. The samples were then lysed with laemelli buffer before being western blotted for $pVASP^{ser159}$, $pMLC^{ser19}$, $pRhoA^{ser188}$, and GAPDH. Images are representative of at least three experiments. **b)** Spreading the platelets as above, they were treated with tyrodes containing 10nM PGI_2 with or without PKA inhibitors 100μM RP-8CPT-cAMP and 2μM KT5720 for a further 10 minutes. The samples were then lysed, before the addition of RhoA GTP beads. Samples were then western blotted for active RhoA and total RhoA. Images are representative of at least three experiments. $p<0.05$.

SUPPLEMENTARY VIDEOS

Supplementary Video 1: Formation of thrombi over fibrinogen coated flow chambers. Whole blood, stained with 10μM $DiOC_6$ was flown over fibrinogen (300μg/ml) coated slides for 2 minutes at a shear rate of $1000s^{-1}$, to enable the formation of thrombi. Videos are representative of three experiments.

Supplementary Video 2: Post perfusion of tyrodes does not affect thrombus embolization on fibrinogen. After 2 minutes of flowing whole blood and formation of thrombi, tyrodes was perfused over these preformed thrombi for 10 minutes at $1000s^{-1}$ and the effect on thrombi was observed. Videos are representative of three experiments.

Supplementary Video 3: Post perfusion of 100nM PGI_2 induces embolisation of preformed thrombi on fibrinogen. After 2 minutes of flowing whole blood and formation of thrombi, tyrodes containing 100nM PGI_2 was perfused over these preformed thrombi for 10 minutes at $1000s^{-1}$ and the effect on thrombi was observed. Videos are representative of three experiments.
